# Inducing effects of cellulosic hydrolysate components of lignocellulose on cellulosome synthesis in *Clostridium thermocellum*

**DOI:** 10.1111/1751-7915.13293

**Published:** 2018-06-25

**Authors:** Renmin Li, Yingang Feng, Shiyue Liu, Kuan Qi, Qiu Cui, Ya‐Jun Liu

**Affiliations:** ^1^ Shandong Provincial Key Laboratory of Energy Genetics CAS Key Laboratory of Biofuels Qingdao Engineering Laboratory of Single Cell Oil Qingdao Institute of Bioenergy and Bioprocess Technology Chinese Academy of Sciences Qingdao China; ^2^ Chinese Academy of Sciences University of Chinese Academy of Sciences Beijing China

## Abstract

Cellulosome is a highly efficient supramolecular machine for lignocellulose degradation, and its substrate‐coupled regulation requires soluble transmembrane signals. However, the inducers for cellulosome synthesis and the inducing effect have not been clarified quantitatively. Values of cellulosome production capacity (CPC) and estimated specific activity (eSA) were calculated based on the primary scaffoldin ScaA to define the stimulating effects on the cellulosome synthesis in terms of quantity and quality respectively. The estimated cellulosome production of *Clostridium thermocellum* on glucose was at a low housekeeping level. Both Avicel and cellobiose increased CPCs of the cells instead of the eSAs of the cellulosome. The CPC of Avicel‐grown cells was over 20‐fold of that of glucose‐grown cells, while both Avicel‐ and glucose‐derived cellulosomes showed similar eSA. The CPC of cellobiose‐grown cells was also over three times higher than glucose‐grown cells, but the eSA of cellobiose‐derived cellulosome was 16% lower than that of the glucose‐derived cellulosome. Our results indicated that cello‐oligosaccharides played the key roles in inducing the synthesis of the cellulosome, but non‐cellulosic polysaccharides showed no inducing effects.

## Introduction

Lignocellulosic biomass is the most abundant renewable carbon resource and has been considered an attractive substitute for fossil sources (Lynd *et al*., 2005; Akinosho *et al*., 2014; Blumer‐Schuette *et al*., 2014). Lignocellulose has a complex and diverse composition, and its deconstruction and bioconversion rely on cellulolytic microorganisms and their cellulases. Thus, a robust and highly active enzymatic system is the precondition of the cost‐effective utilization of lignocellulose (Demain *et al*., 2005; Blumer‐Schuette *et al*., 2014). *Clostridium thermocellum* (recently reclassified as *Ruminiclostridium thermocellum*) is a typical cellulolytic bacterium (Ng *et al*., 1977), and its capability of lignocellulose degradation comes from the extracellular multiprotein complex it produces, termed cellulosome (Bayer *et al*., 2004).

Cellulosome is a highly organized cellulose degrading system containing both enzymatic subunits and non‐catalysing scaffoldins (Bayer *et al*., 1998). Various enzymes with different functions are assembled into scaffoldins by specific interactions between the enzyme‐containing dockerin and the multiple scaffoldin‐bearing cohesin modules. The cellulosome can interact with cellulosic substrates by virtue of the cellulose‐binding modules (CBM), and the complex is anchored to the cell surface via an S‐layer homology (SLH) module on an anchoring scaffoldin (Fontes and Gilbert, 2010; Artzi *et al*., 2017). The resulted multilevel synergy effects endow the cellulosome advantages of cellulose hydrolysis compared to fungus‐derived free cellulases (Johnson *et al*., 1982; Lu *et al*., 2006; Hirano *et al*., 2015; Zhang *et al*., 2017). In addition, the cellulosome of *C. thermocellum* has the superiority for its substrate‐coupled dynamic regulation mechanism (Raman *et al*., 2011; Smith and Bayer, 2013; Wilson *et al*., 2013; Wei *et al*., 2014). That is, *C. thermocellum* can sense the changes of extracellular polysaccharides and regulate the expression of cellulosomal proteins accordingly.

The cellulosomal regulation is mainly mediated by extracytoplasmic function (ECF) sigma factors in *C. thermocellum* (Kahel‐Raifer *et al*., 2010; Nataf *et al*., 2010; Munoz‐Gutierrez *et al*., 2016), and other cellulosome‐ or non‐cellulosome‐related regulatory factors have also been proposed responding to different polysaccharides (Newcomb *et al*., 2007; Wilson *et al*., 2016; Choi *et al*., 2017). In terms of the insolubility of extracellular substrates and the requirement of transmembrane signal transduction, there must be specific soluble compounds performing as signal molecules for the dynamic regulation. Because *C. thermocellum* strains can produce cellulosome using cellobiose as the sole carbon source, cellobiose has been deemed as a key inducer of the cellulosome production (Bhat *et al*., 1993). Previous transcriptomic and proteomic analyses confirmed the expression of cellulosomal proteins in cellobiose, and different compositions were observed for cellobiose‐ and Avicel‐derived cellulosome (Gold and Martin, 2007; Riederer *et al*., 2011; Wei *et al*., 2014; Yoav *et al*., 2017), indicating there might be other inducers besides cellobiose for cellulosomal regulation in *C. thermocellum*.

Besides cellobiose, *C. thermocellum* can grow on glucose after a long adaption phase and produce cellulosomal proteins as well (Yoav *et al*., 2017). Previous bioenergetics analysis indicated that *C. thermocellum* might prefer to assimilate cello‐oligosaccharides with the degree of polymerization of at least four during growth on cellulose (Zhang and Lynd, 2005). Thus, cellulosic hydrolysate components other than cellobiose may also play roles in the induction of the cellulosome synthesis, but their effects have not been extensively analysed quantitatively. Additionally, although non‐cellulosic hydrolysate components cannot be assimilated by *C. thermocellum*, the cellulosome contains various hemicellulases and pectinases for lignocellulose solubilization (Gold and Martin, 2007; Yoav *et al*., 2017), and their effects on cellulosome production have not been revealed. In this study, the effects of cellobiose and Avicel on cellulosome synthesis by *C. thermocellum* DSM1313 were extensively analysed in terms of both quantity and quality using a ScaA‐based estimation method. The inducing effects of other lignocellulose‐related polysaccharides and hydrolysate components were also investigated. The results confirmed the inducing effects of cello‐oligosaccharides on producing the cellulosome by *C. thermocellum* cells, while the non‐cellulosic components showed no inducing effect.

## Results

### The cellulosome synthesis of *C. thermocellum* on glucose was at a low housekeeping level

The growth curves of *C. thermocellum* DSM1313 were first determined with the same amount (5 gl^‐1^) of glucose, cellobiose and Avicel as the sole carbon source. Fig. S1 showed that *C. thermocellum* could utilize glucose after a long adaptation period but was with less biomass, which was consistent with previous reports (Nochur *et al*., 1990; Strobel *et al*., 1995; Rabemanolontsoa *et al*., 2017). The composition of the cellulosome produced by *C. thermocellum* cells grown on glucose has been analysed previously (Yoav *et al*., 2017), but the cellulosome production level was not reported. To investigate the cellulosome produced by the glucose‐grown cells, both scanning electron microscopy (SEM) and hydrolysis assays were performed for the cells grown at early, middle or late exponential phase according to the growth curves (Fig. S1).

**Figure 1:**
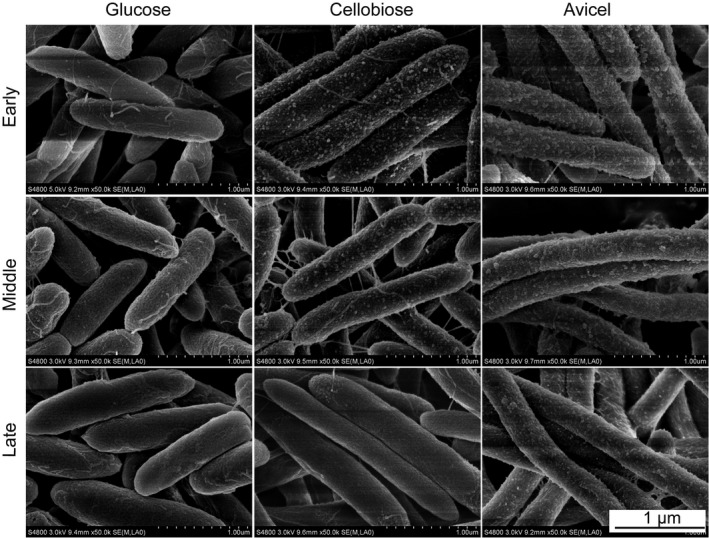
Scanning electron microscopy visualization of *C. thermocellum* cells at early, middle and late exponential phase using glucose, cellobiose or Avicel as the sole carbon source. At the early and middle exponential phase, resting polycellulosomal protuberances are formed on the cell surface with cellobiose or Avicel as the carbon source, and the surface of cells grown on glucose appears smooth but with fibrous structures indicating protuberances in a protracted state. No or few polycellulosomal structures are observed for the cells grown at the late exponential phase because of the detachment of cellulosome from the cell. A scale bar is shown at the bottom right.

**Figure 2:**
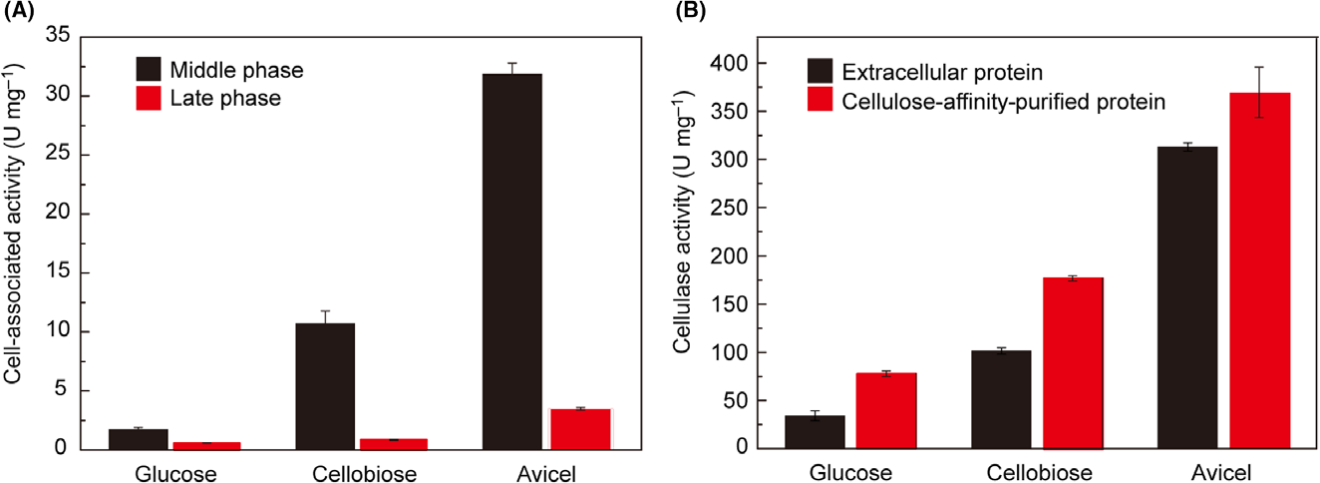
Cellulolytic activities of cell‐attached, cellulose‐affinity‐purified and extracellular proteins of *C thermocellum* with glucose, cellobiose or Avicel as the sole carbon source.A. Cell‐associated hydrolysis assay. The cells at both middle and late exponential stages were used for analysis.B. Cellulose‐affinity‐purified/extracellular protein assay. The values are shown in Table S1. The cells at late exponential stage were used for analysis. The hydrolysis assay with 5 mg Avicel at 55°C for 24 h under oxic conditions, and the amount of reduced sugars was measured using the DNS method. Control reactions containing no Avicel or cells/proteins were prepared, and the amounts of reduced sugars produced by the control reactions were subtracted from the values for the experimental samples. Average values and standard errors indicated by error bars were calculated from three independent experiments

For the cells grown on cellobiose or Avicel, typical resting protuberances referring to the polycellulosomal structures were observed (Bayer and Lamed, 1986), especially at the early and middle exponential phases (Fig. 1). In contrast, the glucose‐grown cells had rather a smooth surface with extended fibrous structures attaching on or connecting to the cells, which is similar to the cell surface morphology of *C. thermocellum* mutants with truncated primary scaffoldin (Hong *et al*., 2014). Therefore, the glucose‐grown *C. thermocellum* cells might synthesize the cellulosome at a very low background housekeeping level. Interestingly, protuberances in both resting and protracted forms could be apparently observed on the surface of cellobiose‐derived cells depending on the growth period (Fig. 1). At the early stage, the cell surface was mainly covered by resting protuberances, while the abundance deceased along with growth, and protracted protuberances increased at the middle stage. At the late growth period, few fibrous structures or resting protuberances were observed on the cell surface (Fig. 1), indicating the detachment of cellulosome from the cell (Bayer *et al*., 1985, 1998; Lamed and Bayer, 1988). These results indicated the synthesis of cellulosomal proteins by *C. thermocellum* with glucose as the sole carbon source, and the glucose‐derived cellulosome might contain different structures compared with that produced from cellobiose and Avicel. Hydrolysis assay was further performed to prove the synthesis of the glucose‐derived cellulosome. The results showed that either cell‐associated, cellulose‐affinity‐purified cellulosomal or extracellular proteins obtained from glucose‐grown cultures had much lower cellulase activity than that produced with cellobiose or Avicel (Fig. 2, Table S1), indicating that the glucose‐derived cellulosome was steadily produced at low level, while the cellulosome synthesis with cellobiose and Avicel was greatly stimulated compared to glucose. According to previous reports, the expression of cellulosomal genes is regulated by both the primary sigma factor SigA and the alternative sigma factor SigI (Nataf *et al*., 2010; Sand *et al*., 2015; Munoz‐Gutierrez *et al*., 2016). The housekeeping SigA is for constitutive cellulosome synthesis at low level, while the environment‐sensitive SigIs induce the high‐level production of the cellulosome. Thus, the low‐level synthesis of glucose‐derived cellulosome observed in this study was consistent with previous studies on cellulosomal regulation.

### Cellulose‐affinity‐purified proteins derived from different substrates contained non‐cellulosomal proteins with diverse abundances

**Figure 3:**
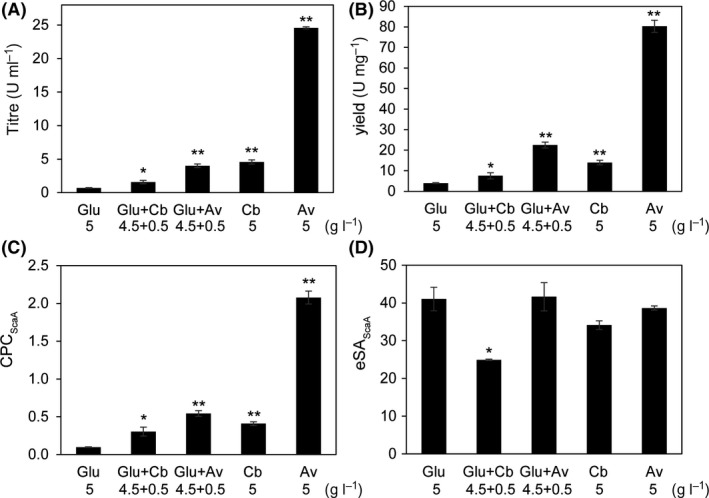
The cellulosomal titre (A), yield (B), estimated production capacity (C) and estimated specific activity (D) of the *C. thermocellum* cells grown on glucose (Glu), cellobiose (Cb) and Avicel (Av) as the carbon sources. The total amount of carbon source was 5 g l^−1^. For inducing experiment, 0.5 g l^−1^ Cb or Av was added in medium containing 4.5 g l^−1^ Glu (Glu + Cb or Glu + Av respectively). The titre and yield were calculated based on the measured protein concentration and specific activity of extracellular proteins (Table S1). The estimated specific activity (eSA_S__caA_) and cellulosomal production capacity (CPC_S__caA_) were calculated based on the proportion of ScaA (pScaA) in the extracellular proteins (Table S1). Three independent analyses were performed for statistical calculation. *P*‐value was calculated using Student's t‐test with Glu as the reference. *P <* *0.05, **P <* *0.01

To evaluate the effect of cellobiose and Avicel on the cellulosome synthesis in *C. thermocellum*, the titre and yield of the cellulosome were calculated based on the following equations:

$${\text{titer}}_{e}=S_{e}\times C_{e};{\;\text{titer}}_{\mathrm{cp}}=S_{\mathrm{cp}}\times C_{\mathrm{cp}}$$

$${\text{yield}}_{e}=\frac{{\;\text{titer}}_{e}}{C_{p}};\;{\text{yield}}_{\mathrm{pc}}=\frac{{\;\text{titer}\;}_{\mathrm{cp}}}{C_{p}}$$

in which titre (U ml^−1^) describes the hydrolysis activity per ml culture and yield (U mg^−1^) describes the hydrolysis capability per mg pellet protein (to represent the cells). *S* indicates the specific activity (U mg^−1^) of the extracellular (*S*_e_) or cellulose‐affinity‐purified (*S*_cp_) proteins (Table S1), and C indicates the protein concentration (mg ml^−1^) of the extracellular (*C*_e_), cellulose‐affinity‐purified (*C*_cp_) or pellet (*C*_p_) proteins in 100‐ml culture (Table S1). The results indicated that both titre and yield of the cellulose‐affinity‐purified proteins were greatly stimulated by either cellobiose or Avicel compared to that of glucose‐derived cellulosome (Fig. 3A, B).

To evaluate whether the cellulosomal components were efficiently purified from the extracellular fractions using the cellulose‐affinity method, the titre and yield values were calculated based on both extracellular protein and cellulose‐affinity‐purified proteins. For glucose, the calculated values showed a slight difference. However, significant changes were observed when cellobiose or Avicel was used as the carbon source. The values calculated based on cellulose‐affinity‐purified proteins were 42% and 27% lower than those calculated based on extracellular proteins for cellobiose and Avicel respectively (Fig. S2). The reduced values might be caused by the great loss of true cellulosomal proteins, the disturbance of unexpectedly purified non‐cellulosomal portions, or the dramatic contribution of non‐cellulosomal proteins to cellulose hydrolysis. As the cellulosome has been considered the major enzymatic system for cellulose degradation in *C. thermocellum* (Xu *et al*., 2016; Yoav *et al*., 2017), the contradictory results indicated that the present cellulosomal purification method might not be with high efficiency.

**Figure 4:**
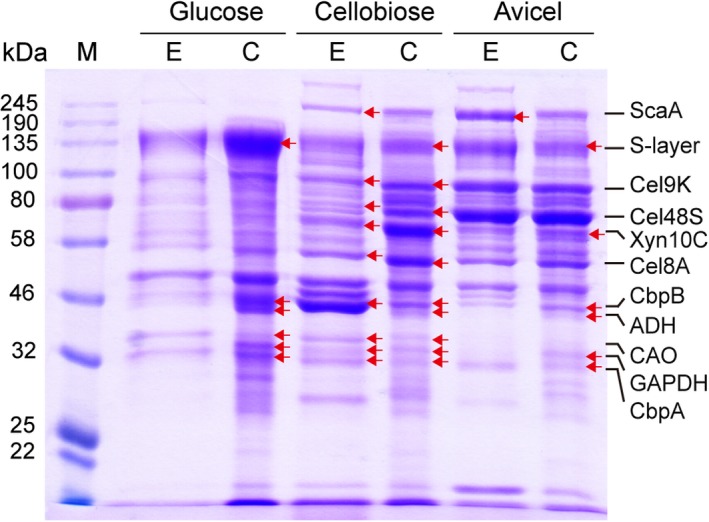
SDS‐PAGE analysis of extracellular (E) and cellulose‐affinity‐purified (C) proteins of *C. thermocellum* cultivated with glucose, cellobiose or Avicel as the sole carbon source. Protein bands (red arrows) with abundance changes among different samples were selected for mass spectroscopy identification as shown to the right of the Coomassie blue‐stained gel. Besides cellulosomal proteins (ScaA, Cel48S, Cel9K, Cel8A, Xyn10C), several non‐cellulosomal proteins were detected, including S‐layer (S‐layer homology protein, Clo1313_RS15300), CbpB (sugar ABC transporter substrate‐binding protein, Clo1313_RS06075), ADH (alcohol dehydrogenase, Clo1313_RS09240), CAO (copper amine oxidase‐like protein, Clo1313_RS11630), GAPDH (glyceraldehyde‐3‐phosphate dehydrogenase, Clo1313_RS10615) and CbpA (sugar ABC transporter substrate‐binding protein Clo1313_RS09245). M, protein standards.

The compositions of the cellulose‐affinity‐purified proteins and extracellular proteins from different carbon sources were further analysed by SDS‐PAGE and MS to evaluate the purification specificity. The results showed that the cellulose‐affinity‐purified proteins might contain several non‐cellulosomal proteins, including S‐layer homology protein (Clo1313_RS15300), cellodextrin transporters (Clo1313_RS09245, Clo1313_RS06075), alcohol dehydrogenase (Clo1313_RS09240), copper amine oxidase‐like protein (Clo313_RS11630) and glyceraldehyde‐3‐phosphate dehydrogenase (Clo1313_RS10615) (Fig. 4). The composition of the cellulose‐affinity‐purified proteins was further determined by analysing the protein electrophoresis gel using the Quantity One Software (BioRad). The glucose‐derived samples contained a higher abundance of non‐cellulosomal proteins compared to cellobiose‐ or Avicel‐derived samples (Fig. 4, Table S2). This result indicated the non‐specific purification of non‐cellulosomal proteins, and the low‐level synthesis of glucose‐derived cellulosome could aggravate the non‐specific purification.

Thus, to determine the activity, production and composition of the cellulosome derived from different carbon sources, the cellulosomal proteins should be precisely determined and quantified to eliminate the disturbance of non‐cellulosomal portions. However, it is difficult to separate cellulosomal components from other extracellular proteins using regular chromatography‐based purification procedures because non‐cellulosomal proteins may be purified simultaneously through the unspecific protein–protein interactions with cellulosomal components, which may result in the underestimation of the cellulosomal activity and the overrating of the cellulosomal production. Nevertheless, the specific activity and the amount of the cellulosome may be estimated based on the primary scaffoldin ScaA (also known as CipA).

### Both Avicel and cellobiose stimulate the cellulosomal production capability of the cells

ScaA is the basal component for cellulosomal assembly in *C. thermocellum* (Zverlov *et al*., 2008; Olson *et al*., 2013; Yoav *et al*., 2017) and was proposed to represent the cellulosome (Dykstra *et al*., 2014). The protein electrophoresis gels were analysed by the Quantity One Software to calculate the proportion of ScaA (pScaA) in the total extracellular proteins under various conditions. A strong linear correlation between pScaA and the hydrolysis activity of extracellular proteins was observed with an *R* ^*2*^ value of 0.979 (Fig. S3). Hence, it is possible to estimate the production and specific activity of the cellulosome in terms of quantity and quality, respectively, based on the following equations:

$${\text{eSA}}_{\mathrm{ScaA}}\;\overset{\mathrm{def}}{=}\;\frac{S_{e}}{\text{pScaA}}$$

$${\text{CPC}}_{\mathrm{ScaA}}\;\overset{\mathrm{def}}{=}\;\frac{\text{pScaA}\times C_{e}}{C_{p}}$$

in which eSA_ScaA_ is calculated using the parameter pScaA and the determined specific activity (*S*_e_) of the extracellular proteins (Table S1) to describe the estimated specific activity of the cellulosome based on ScaA; CPC_ScaA_ is calculated using pScaA and the concentrations of the extracellular (*C*_e_) and pellet proteins (*C*_p_) (Table S1) to describe the estimated cellulosome production capability of the cells based on ScaA (i.e. the amount of cellulosomal proteins that can be produced per unit biomass).

Fig. 3C showed that CPC_ScaA_ for cellobiose or Avicel was 4.19 or 21.26 times of that for glucose, respectively, indicating the significantly stimulated CPC of the cells. The addition of a low amount of cellobiose or Avicel (0.5 g l^−1^) in the glucose medium also showed the stimulating effect on the CPC, but with less extent. Similar eSA values were detected for the cellulosome produced from Avicel and glucose, indicating that Avicel did not influence the cellulosomal activity (Fig. 3D). However, lower eSA was detected when cellobiose was used as the carbon source, and the change became significant when 0.5 g l^−1^ cellobiose was supplemented (Fig. 3D). This result indicated cellobiose mainly stimulated the ability of the cells to produce cellulosome, but not the quality of the produced cellulosome.

### Cello‐oligosaccharides could induce the synthesis of cellulosomal protuberances by *C. thermocellum*

**Figure 5:**
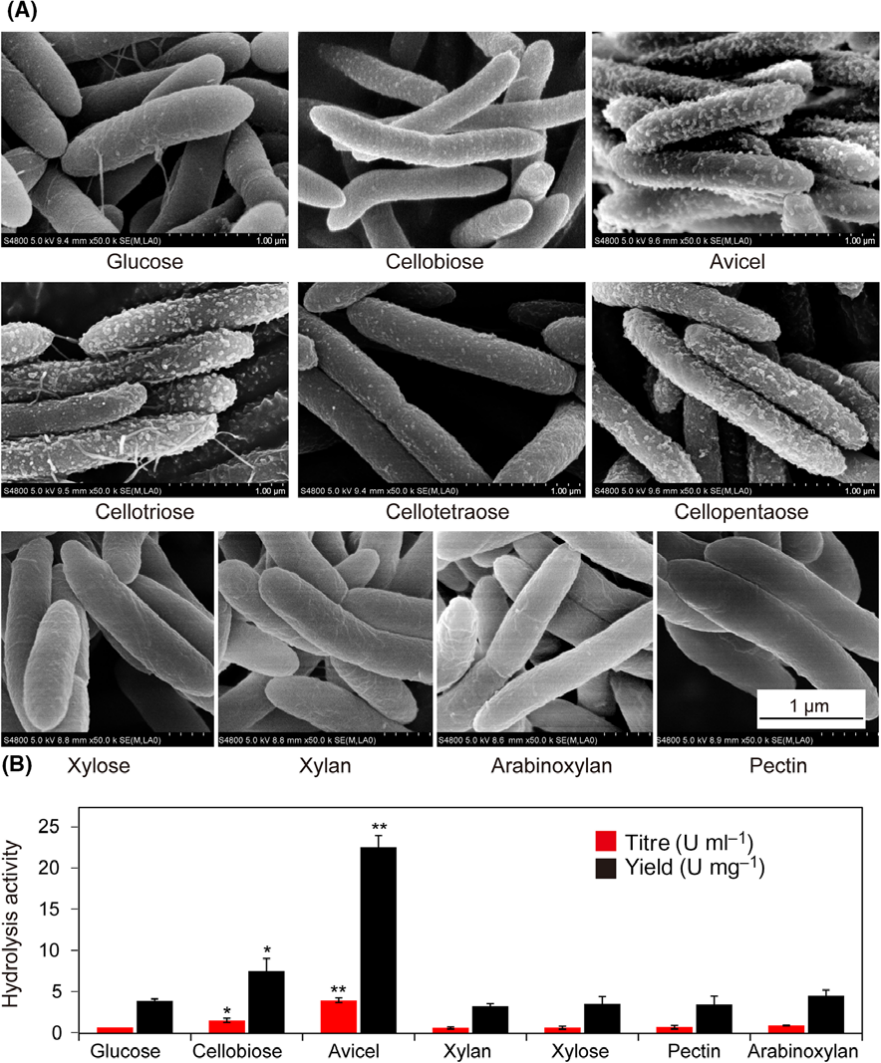
Scanning electron microscopy visualization (A) and hydrolysis activity (B) of *C. thermocellum* cells grown on glucose supplemented with 0.5 g l^−1^ various carbon sources. The total amount of carbon source is 5 g l^−1^.A. Polycellulosomal protuberances could be observed on the cell surface when cellobiose, cellotriose, cellotetraose, cellopentaose or Avicel was supplemented, while no protuberances were observed for xylan, pectin, arabinoxylan or xylose. Scale bar is shown at the bottom right.B. The extracellular hydrolysis activities of the *C. thermocellum* cells. The titre and yield values were calculated based on the measured protein concentration and specific activity of extracellular proteins (Table S1). *P*‐values were calculated to determine using Student's t‐test with glucose as the reference. *P <* *0.05, **P <* *0.01.

The eSA of the cellobiose‐derived cellulosome is not as high as that derived from glucose or Avicel, indicating that there should be other lignocellulose‐related hydrolysate components performing as the transmembrane inducers besides cellobiose for the maintaining of the cellulosomal quality. Clear protuberance structures were observed on the cell surface of *C. thermocellum* by SEM when 0.5 g l^−1^ cellobiose was present in the medium, indicating that low amount of cellobiose could induce the synthesis of the cellulosome (Fig. S4). The inducing effects of selected lignocellulosic polysaccharides (xylan, pectin and arabinoxylan) and hydrolysate components (xylose, cellotriose, cellotetraose and cellopentaose) were further tested with cellobiose and Avicel as the positive controls at the same concentration of 0.5 g l^−1^ at early exponential phase. SEM analysis showed polycellulosomal protuberances on the cell surface when cellotriose, cellotetraose or cellopentaose was supplemented as the inducer. For xylan, pectin, arabinoxylan or xylose, no protuberances were observed (Fig. 5A), and slight or no change in the titre or yield of the cellulose‐affinity‐purified cellulosome was detected (Fig. 5B). These non‐cellulosic substrates also showed slight influence on the composition of extracellular proteins (Fig. S5). This result suggested that low concentration of cellulose hydrolysate components could induce the cellulosome synthesis, but non‐cellulose components of lignocellulose showed no inducing effects.

## Discussion

In this study, we analysed and estimated the effects of cellobiose and Avicel on cellulosome synthesis by *C. thermocellum* in terms of quantity and quality. Our results confirmed the stimulating effects of cellobiose and Avicel on the cellulosome production capability instead of the estimated cellulosomal specific activity. The inducing effects of other lignocellulose‐related polysaccharides and hydrolysate components were also investigated, and the results indicated that non‐cellulosic components could not induce the synthesis of the cellulosome. In addition, we employed an MS‐analysis‐independent method to estimate the production and specific activity of the cellulosome based on the proportion of the primary scaffoldin ScaA (pScaA) in the total extracellular proteins. Our results indicated the *pScaA*‐based method was simple, convenient and practical.

Cellulosome has been determined as one of the most effective supramolecular machines for cellulose degradation in nature, and its functionality has been extensively studied to reveal its efficient mechanism (Fontes and Gilbert, 2010; Artzi *et al*., 2017; Smith *et al*., 2017). Cellulosome contains both enzymatic subunits and non‐catalysing scaffoldin proteins, and the importance of proper composition and enzyme–scaffoldin ratios for the cellulosic activity of the cellulosome have been analysed extensively (Zverlov *et al*., 2008; Saharay *et al*., 2010; Olson *et al*., 2013; Hong *et al*., 2014; Yoav *et al*., 2017), as well as the resulted synergetic effects (Johnson *et al*., 1982; Lu *et al*., 2006; Hong *et al*., 2014; Hirano *et al*., 2015). Several studies have shown that the cellulosome of *C. thermocellum* could adjust the composition and enzyme abundance along with the extracellular substrates dynamically (Dror *et al*., 2003; Gold and Martin, 2007; Raman *et al*., 2009, 2011), and ECF‐like sigma–antisigma (SigI‐RsgI) factors are considered to be responsible for the cellulosomal regulation in *C. thermocellum* (Kahel‐Raifer *et al*., 2010; Nataf *et al*., 2010; Munoz‐Gutierrez *et al*., 2016). Using SigI‐RsgI factors, *C. thermocellum* can sense the change of extracellular polysaccharides and regulates the expression of relative cellulosomal genes accordingly. However, the SigI‐RsgI regulation mechanism requires further illustration, including the identification of the transmembrane signals.

The inducing effect of cellobiose on cellulase synthesis has been considered a general phenomenon in cellulase‐producing fungi, such as *Penicillium decumbens*,* Penicillium oxalicum, Hypocrea jecorina* and *Neurospora crassa* (Zhou *et al*., 2012; Znameroski *et al*., 2012; Chen *et al*., 2013; Li *et al*., 2017). As the main product of the cellulosome system, cellobiose has also been considered to behave as a transmembrane signal to induce the production of the cellulosome in *C. thermocellum* strains (Bhat *et al*., 1993). However, inconsistent compositions of cellobiose‐ and Avicel‐derived cellulosomes have been observed (Gold and Martin, 2007; Riederer *et al*., 2011; Wei *et al*., 2014; Yoav *et al*., 2017), which indicated that there might be other inducers for cellulosome synthesis. In addition, Yoav *et al*. observed the lower specific activity of cellobiose‐derived cellulosome compared to Avicel‐derived cellulosome caused by the distinguished cellulosomal composition (Yoav *et al*., 2017), but the inducing effects of cellobiose and Avicel on cellulosome production have not been analysed quantitatively.

We first considered the cellulose‐affinity‐purified fractions as the cellulosomal proteins, using which the values of titre and yield were calculated for *C. thermocellum* with cellobiose and Avicel as the sole carbon source to describe the hydrolysis activity per ml of the cell culture and per cell dry weight (represented by the pellet protein) respectively. However, although both extracellular or cellulose‐affinity‐purified protein‐based values of cellobiose or Avicel were significantly higher than those of glucose, which confirmed the stimulating effect of cellobiose and Avicel on synthesizing the cellulosome, the differences between the extracellular and cellulose‐affinity‐purified protein‐based values were significant. This result indicated a non‐specific extraction of non‐cellulosomal fractions in cellulose‐affinity‐purified proteins. Further SDS‐PAGE and MS analyses confirmed that the purified proteins contained several non‐cellulosomal proteins. Thus, the cellulose‐affinity‐purified proteins could not represent the true cellulosomal proteins and should not be directly used to analyse and compare cellulosomal activity, abundance and composition. Higher abundance of non‐cellulosomal proteins was detected for the glucose‐derived samples, indicating that low‐level synthesis of the cellulosome might aggravate the non‐specific affinity of non‐cellulosomal proteins to cellulose.

Previous studies purified the cellulosome from the concentrated supernatants of the culture using size exclusion chromatography (Artzi *et al*., 2015; Yoav *et al*., 2017). Different fractions with cellulase activity were pooled together as the true cellulosomal proteins, and MS analysis was further performed to separate cellulosomal proteins from non‐cellulosomal proteins. In this way, the contamination of non‐cellulosomal proteins would be avoided to a large extent, and the true cellulosomal activity could be calculated. Thus, the precise identification and calculation of the true cellulosomal components were based on the proteomic method so far. Because ScaA is the primary assembly subunit of the cellulosome and its abundance showed linear correlation with the hydrolysis activity of extracellular proteins as well as the total cellulosomal mass concentration (Dykstra *et al*., 2014), it may be used to estimate the specific activity and abundance of cellulosomal fractions. Because the extracellular proteins of *C. thermocellum* at the exponential stage involve the majority of cellulosomal proteins and the hydrolysis activity of extracellular proteins is mostly from cellulosomal proteins, a ScaA‐based estimation method was developed in this study to compare the produced cellulosomes from various substrates in terms of quantity and quality. According to the ScaA‐based estimation, cells grown on both Avicel and cellobiose showed higher ability in producing cellulosome compared to those grown on glucose, but cellobiose‐derived cellulosome had lower eSA. This conclusion was supported by previous MS‐based study, and the reduced specific activity of the cellobiose‐derived cellulosome might be caused by the change of the cellulosomal composition (Yoav *et al*., 2017). Thus, *C. thermocellum* cells might regulate the ratio of cellulosomal components towards lower cellulosic activity by sensing its preferred substrate cellobiose. Additionally, the decreasing degree of eSA became significant with the addition of 0.5 g l^−1^ instead of 5 g l^−1^ cellobiose, indicating that the repressing effect of cellobiose might be dose‐dependent. The cellobiose‐repression effect should be considered in further study on cellulosomal regulation.

It is noteworthy that the ScaA‐based estimation method has limited accuracy in the determination of the cellulosomal activity and abundance. The method works well only when two following prerequisites are satisfied: (i) the cellulosome‐producing bacterium harbours single primary scaffoldin and (ii) the cellulosomes produced under different conditions contain the primary scaffoldin and similar enzymatic components at average level. The first prerequisite is obviously satisfied for *C. thermocellum* analysed in this study, but not for *Acetivibrio cellulolyticus* (Hamberg *et al*., 2014), *Pseudobacteroides cellulosolvens* (Zhivin *et al*., 2017), etc., which have more complex cellulosomal architectures. The second prerequisite is likely true in this study because both glucose and cellobiose are the hydrolysates of Avicel. Therefore, these three substrates may induce cellulosome synthesis through a consistent pathway and results in the same, or very similar, enzymatic cellulosomal components. The ScaA‐based estimation method may have problems when the cellulosomes derived from complex lignocellulosic substrates are compared to Avicel‐derived cellulosome, because non‐cellulosic components may influence the cellulosomal composition and result in a change of the average molecular weight of the cellulosomes.

*Clostridium thermocellum* can grow with glucose as the sole carbon source and produce cellulosome steadily although at a low level. The glucose‐derived cellulosome might be produced at the housekeeping level to ensure that the cells can quickly respond to extracellular polysaccharides. Although the function of glucose on cellulosome synthesis is not clear so far, it should be considered an important carbon source for further study on cellulosomal regulation. *C. thermocellum* has eight pairs of SigI‐RsgI factors those have been proposed to be mainly responsible for cellulosomal regulation (Nataf *et al*., 2010; Munoz‐Gutierrez *et al*., 2016). Each RsgI contains a C‐terminal extracellular domain, such as CBMs (CBM3, CBM42), sugar‐binding elements (PA14) and glycoside hydrolases (GH10, GH5) that have polysaccharide‐related functions, in which CBM3 and CBM42 mainly sense and interact with cellulose and arabinoxylan respectively. PA14 can strongly interact with pectin, and GH10 and GH5 are involved in the hydrolysis of cellulose and xylans (Kahel‐Raifer *et al*., 2010; Bahari *et al*., 2011). Thus, the synthesis of cellulosomal units might be stimulated by xylan, arabinoxylan and pectin besides cellulose. Nevertheless, the addition of selected non‐cellulose‐related polysaccharides or monosaccharide showed no or slight effects on the cellulosome synthesis, which was indicative of the roles of cellulosic hydrolysate components as the main inducers of the cellulosome in *C. thermocellum*. This implies the cross‐talk between different pairs of SigI‐RsgI factors, and the cellulose‐sensing SigI‐RsgI factors are prerequisite for high‐level cellulosome production.

The SigI‐RsgI regulating mechanism has been proposed. Based on which, the extracellular domain of RsgI interacts with cellulosic substrates which may induce a conformational change on the intracellular domain, resulting in the release of SigI for initiating the gene transcription (Nataf *et al*., 2010). In this study, we observed that the cellulosic hydrolysates showed different inducing effects on the synthesis of the cellulosome. For example, cellobiose did not stimulate the estimated specific activity of the cellulosome as Avicel did. Lower abundance of protuberances on the surface of cellotetraose‐grown cells was detected compared to those grown on cellotriose, cellopentanose and Avicel (Fig. 5A). Thus, besides the extracellular substrate‐induced conformation change, the cellulosic hydrolysates‐mediated induction is an essential mechanism for cellulosomal regulation as well. Additionally, as the preferred assimilating cellulosic substrate of *C. thermocellum* (Zhang and Lynd, 2005), cellotetraose might be a key cellulosic hydrolysate for regulating the cellulosomal composition.

## Experimental procedures

### Bacterial strains and cultivation

*Clostridium thermocellum* DSM1313 was cultured at 55°C in 250‐ml anaerobic bottles in 100‐ml modified GS‐2 medium (Cui *et al*., 2012) with 5 g l^−1^ glucose, cellobiose or Avicel (Sigma PH101) as the carbon source. 0.5 g l^−1^ cellobiose, cellotriose, cellotetraose, cellopentaose, Avicel, xylose, xylan, arabinoxylan or pectin was added in the medium with 4.5 g l^−1^ glucose for inducing experiment. In detail, the cells are first cultured with cellobiose as the sole carbon source until the exponential phase, harvested by centrifugation, washed two to three times with modified GS‐2 medium without carbon source, and then inoculated into glucose‐, cellobiose‐ or Avicel‐containing media. During the cultivation, 2‐ml cultures were sampled every 4–12 h with a 2.5‐ml syringe, and rested in an ice bath for 20 min to settle the cellulose residue, if any. Then, 1‐ml cell suspension of the samples was carefully transferred to another tube and centrifuged to obtain the cells, which were used to determine the growth curve by analysing pellet proteins. Three biological replicates were set up for each condition.

### Preparation of extracellular and pellet proteins

*Clostridium thermocellum* cells were cultivated to the late exponential stage with various carbon sources. The cultures were deposited in an ice bath for 20 min to settle the cellulose residue, if any, to the bottle bottom while the cells were still suspended in the supernatant. The cell suspensions were carefully sampled using a 5‐ml syringe without shake and concentrated at 3000 *g* for 30 min to separate supernatant from the pellets. For the supernatant, 10 ml was further 20 times concentrated using Amicon Ultra‐15 centrifugal filter units (10.0 kDa cut‐off) (Merck Millipore, Shanghai, China) as the extracellular proteins, and the rest (50–80 ml) was used for cellulose‐affinity purification. The pellets were used to determine the cell‐associated activity and pellet protein concentrations. The pellet proteins were analysed by boiling the cells in 0.5‐ml 0.2 M NaOH for 10 min and centrifugation (8000 *g*, 10 min) according to a previous procedure (Hong *et al*., 2014), and the concentration of pellet proteins was quantified by the Bradford method (Bradford, 1976).

### Cellulose‐affinity purification

Cellulose‐affinity purification was performed according to a previously reported procedure (Morag *et al*., 1992). In detail, the supernatants were incubated at 4°C overnight with 10 mg phosphoric acid‐swollen amorphous cellulose and then centrifuged at 3000 *g* for 30 min at 4°C. The obtained pellets were resuspended with 10 ml 50 mM Tris–HCl buffer (pH 7.0) containing 10 mM CaCl_2_ and 5 mM dithiothreitol, and then dialysed against 1 l of sterile distilled water until the phosphoric acid‐swollen amorphous cellulose was no longer reduced. After the digestion, the reaction mixtures were microfiltrated (polyethersulfone syringe filter with a pore size of 0.22 μm), concentrated to 0.5–1 ml by ultrafiltration (10.0 kDa), and quantified by the Bradford method (Bradford, 1976) for further analyses.

### Protein analyses

All extracellular or cellulose‐affinity‐purified protein samples were analysed by sodium dodecyl sulfate‐polyacrylamide gel electrophoresis (SDS‐PAGE). The molecular weight of the protein was estimated according to the relative mobility of protein ladders (11–245 kDa; New England BioLabs, Beijing, China). Mass spectroscopy was used to identify individual protein bands by an LTQ‐ESI‐MS/MS (Thermo Finnigan, Silicon Valley, CA, USA) using a surveyor high‐performance liquid chromatography (HPLC) system equipped with a C18 RP column (0.18 × 100 mm, Thermo Electron Corporation) according to a published procedure (Hong *et al*., 2014). The relative quantity of ScaA in the prepared protein samples was determined using the Quantity One Software (version 4.6.2; BioRad, Shanghai, China). In detail, the lanes in the gel images were first manually defined, and the lane background was subtracted using a rolling disc method with a subtraction size of 100 for all lanes. Then, the bands were automatically identified using the volume rectangle detection method with default parameters. The volumes of the bands are calculated by multiplying the sum of the intensities of the pixels inside the volume boundary with pixel area. The bands were manually added, adjusted or removed when necessary. The relative quantity calculation was performed automatically to define the relative quantity of defined bands in lanes using the calculation method of ‘% of Bands in Lane’. The band with a size between 190 and 245 kDa was determined as ScaA according to a previous study, in which the band with the same size was confirmed as ScaA by mass spectroscopy (Hong *et al*., 2014).

### Scanning electron microscopy

Scanning electron microscopy (SEM) was performed with *C. thermocellum* cells grown at early, middle or late exponential phase with various carbon sources using a field emission scanning electron microscope (S‐4800; Hitachi, Tokyo, Japan) according to previously reported method (Hong *et al*., 2014).

### Hydrolysis assay

The cell‐associated cellulase activity of *C. themocellum* cells was determined under oxic conditions as previously described (Hong *et al*., 2014) using cells growing at middle or late exponential phase. The cells were harvested after sedimentation in an ice bath for 20 min, washed and resuspended in reaction buffer (20 mM acetate, 10 mM CaCl_2_, 5 mM L‐cysteine, and 2 mM EDTA, pH 5.0) and was normalized based on the concentrations of pellet proteins (Hong *et al*., 2014). One milliliter of reaction mixtures containing 20 μl cell suspension and 5 g l^−1^ Avicel were incubated at 55°C for 24 h on a rotary shaker at 170 rpm. Reactions without Avicel or cell suspension were prepared as the negative control, and the amounts of reduced sugars produced by the control reactions were subtracted from the values for the experimental samples. The hydrolysis activities of cellulosomal and extracellular proteins were tested in 1‐ml reaction mixtures with 30–50 μg proteins and 5 mg Avicel as the substrate. The reaction buffer contained 50 mM sodium acetate, 10 mM CaCl_2_ and 5 mM dithiothreitol (DTT), pH 5.5. The reaction mixtures were incubated at 55 °C for 24 h. The produced reducing sugars were measured by the 3,5‐dinitrosalicylic acid (DNS) method (Miller, 1959). One unit of cellulase activity is defined as the amount of proteins that releases 1 μmol reducing sugar (glucose equivalent) per min.

## Conflict of interest

None declared.

## Supporting information


**Figure S1.** The growth curves of *C. thermocellum* DSM1313 cultivated using glucose, cellobiose or Avicel as the sole carbon source. The pellet proteins of the cells were quantified to represent the cell density along with the growth. Three independent replicates were set up for each condition. Arrows above the curves indicate the sampling points at early, middle and late exponential phase for further experiments.

**Figure S2.** The cellulosomal titer (a) and yield (b) of the *C. thermocellum* cells grown on 5 g/L glucose, cellobiose and Avicel as the carbon sources. The values were calculated based on the measured protein concentrations and specific activities of extracellular (black bars) or cellulose‐affinity purified proteins (red bars) (Table S1). Three independent analyses were performed for statistical calculation. *p* values were calculated to determine the influence of carbon sources (cellobiose/ Avicel vs. glucose, *p <* *0.05, **p <* *0.01) or to determine whether the extracellular or cellulose‐affinity purified proteins‐based analysis would affect the titer and yield values (red bars vs. black bars, #*p *<* *0.05, ##*p *<* *0.01).

**Figure S3.** The relationship between the proportion of ScaA in the total extracellular proteins (*pScaA*) and the hydrolysis activity of extracellular proteins (*S*_*e*_) using different carbon sources, including glucose (Glu), cellobiose (Cb), Avicel (Av), xylan (Xyn), xylose (Xyl), pectin (Pt), and arabinoxylan (Ax) as labelled on the figure. The linear equation and *R* ^2^ value are shown. *S*_*e*_ values were calculated based on three independent replicates, and the standard errors are indicated by the vertical error bars. *pScaA* values were calculated based on three independent protein electrophoresis gels or one gel with a protein mixture of three replicates, and the standard errors are indicated by the horizontal error bars when possible.

**Figure S4.** Scanning electron microscopy visualization of *C. thermocellum* cells grown on glucose supplemented with 0 to 1 g/L cellobiose. The total amount of carbon source is 5 g/L. Cells grown on 5 g/L glucose or cellobiose were used as the negative or positive control, respectively. Polycellulosomal protuberance structures could be observed on the cell surface of *C. thermocellum* when 0.5 g/L cellobiose was present in the medium. A scale bar is shown at the bottom right.

**Figure S5.** SDS‐PAGE analysis of extracellular proteins of *C. thermocellum* cultivated with glucose (Glu) as the carbon sources. 0.5 g/L cellobiose (Cb), Avicel (Av), xylan (Xyn), xylose (Xyl), pectin (Pt), or arabinoxylan (Ax) was supplemented as the inducer. M, protein standards.

**Table S1**. The specific activity, concentration and ScaA proportion of the proteins produced by *C. thermocellum* cells grown on different carbon sources.

**Table S2.** The proportion (%) of the MS identified non‐cellulosomal proteins in extracellular proteins and cellulose‐affinity purified proteins of *C. thermocellum* cells grown on different carbon sources.
