# Supplementary figures and images for: Inducing effects of cellulosic hydrolysate components of lignocellulose on cellulosome synthesis in Clostridium thermocellum
Source: Microb Biotechnol. 2018 Jun 25;11(5):905–16. doi: 10.1111/1751-7915.13293 (PMC6116742; doi:10.1111/1751-7915.13293)

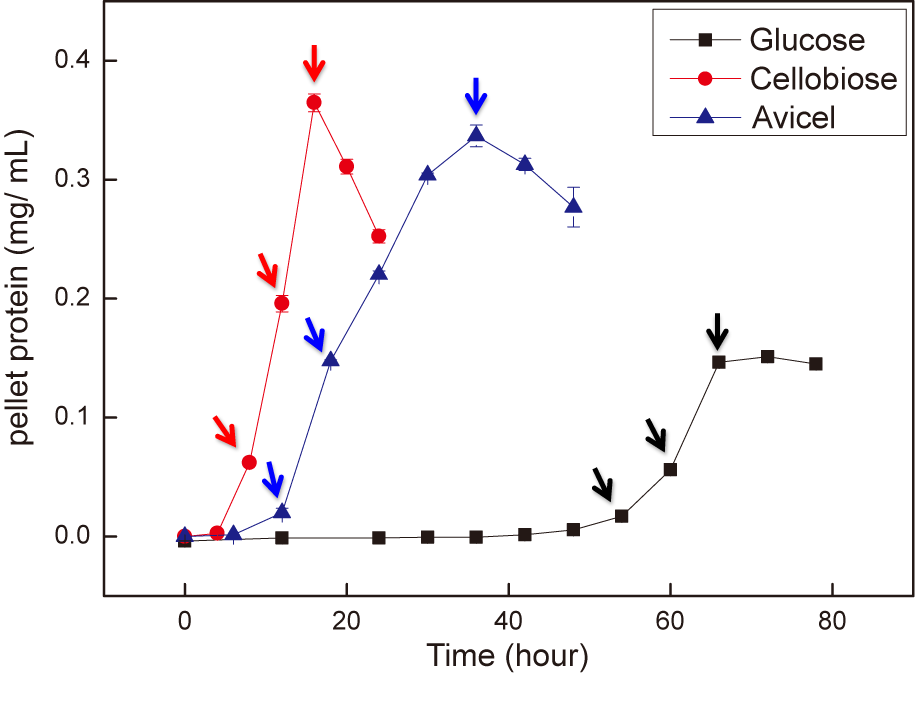

Supplement: Supplementary file 1 — Figure S1. The growth curves of C. thermocellum DSM1313 cultivated using glucose, cellobiose or Avicel as the sole carbon source. The pellet proteins of the cells were quantified to represent the cell density along with the growth. Three independent replicates were set up for each condition. Arrows above the curves indicate the sampling points at early, middle and late exponential phase for further experiments. [file MBT2-11-905-s001.tif]

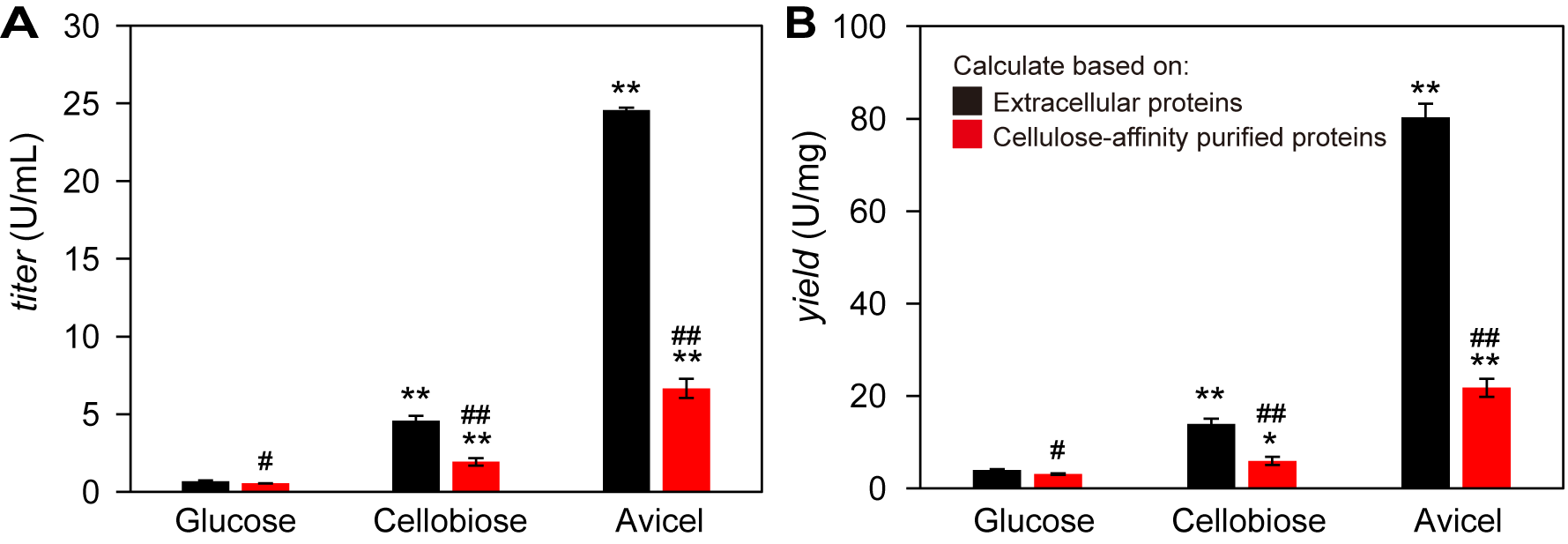

Supplement: Supplementary file 2 — Figure S2. The cellulosomal titer (a) and yield (b) of the C. thermocellum cells grown on 5 g/L glucose, cellobiose and Avicel as the carbon sources. The values were calculated based on the measured protein concentrations and specific activities of extracellular (black bars) or cellulose‐affinity purified proteins (red bars) (Table S1). Three independent analyses were performed for statistical calculation. p values were calculated to determine the influence of carbon sources (cellobiose/ Avicel vs. glucose, *p < 0.05, **p < 0.01) or to determine whether the extracellular or cellulose‐affinity purified proteins‐based analysis would affect the titer and yield values (red bars vs. black bars, #p < 0.05, ##p < 0.01). [file MBT2-11-905-s002.tif]

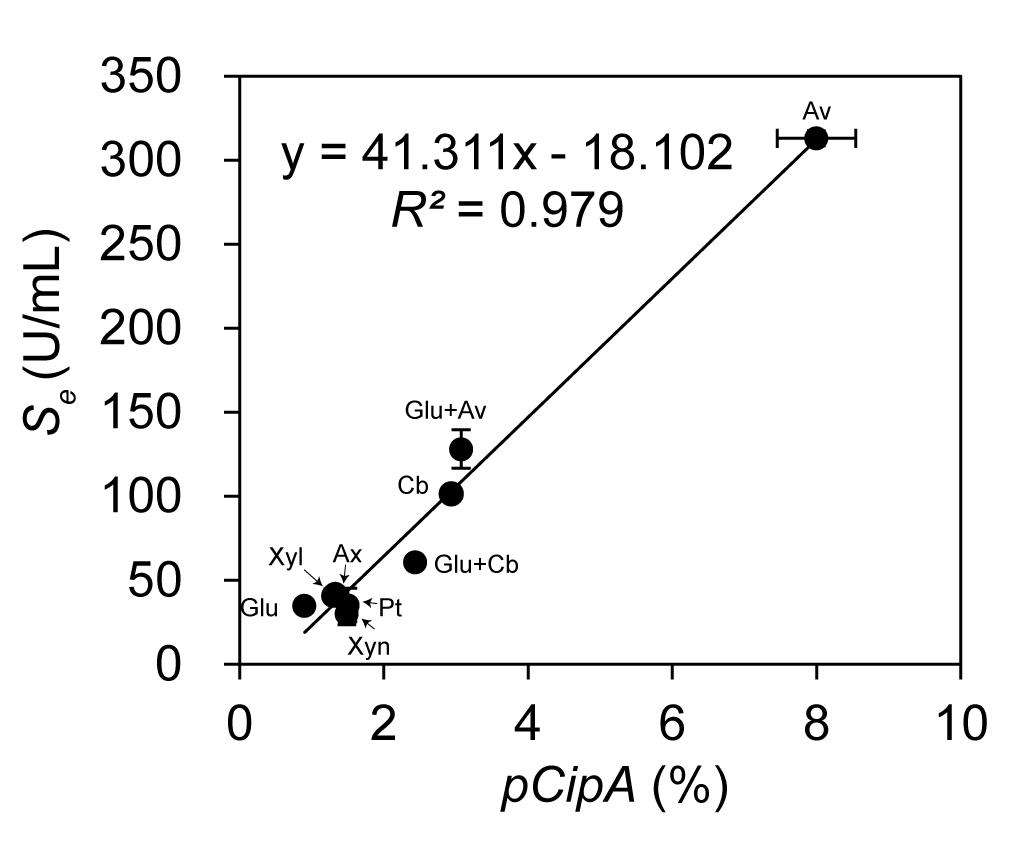

Supplement: Supplementary file 3 — Figure S3. The relationship between the proportion of ScaA in the total extracellular proteins (pScaA) and the hydrolysis activity of extracellular proteins (S e) using different carbon sources, including glucose (Glu), cellobiose (Cb), Avicel (Av), xylan (Xyn), xylose (Xyl), pectin (Pt), and arabinoxylan (Ax) as labelled on the figure. The linear equation and R 2 value are shown. S e values were calculated based on three independent replicates, and the standard errors are indicated by the vertical error bars. pScaA values were calculated based on three independent protein electrophoresis gels or one gel with a protein mixture of three replicates, and the standard errors are indicated by the horizontal error bars when possible. [file MBT2-11-905-s003.tif]

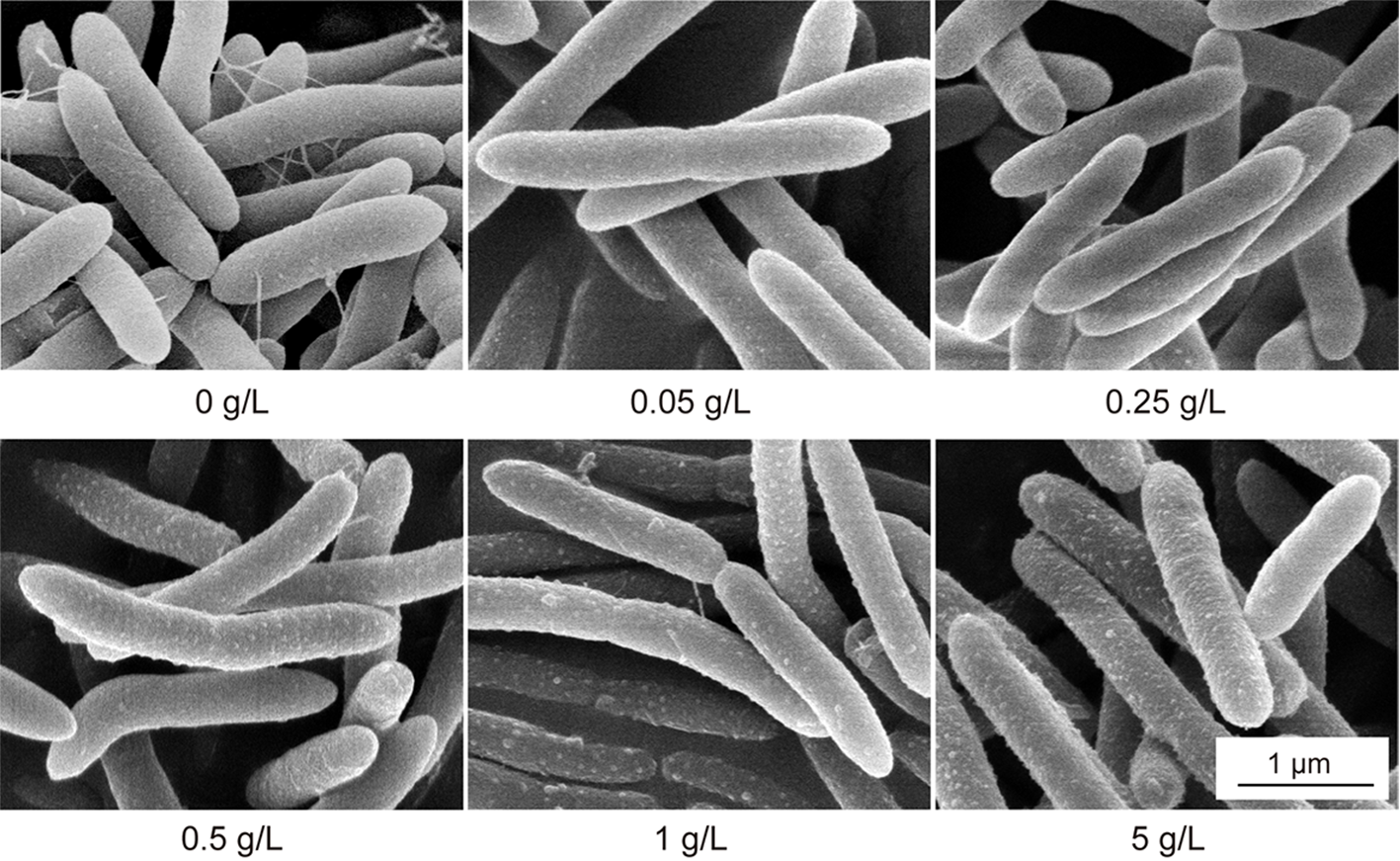

Supplement: Supplementary file 4 — Figure S4. Scanning electron microscopy visualization of C. thermocellum cells grown on glucose supplemented with 0 to 1 g/L cellobiose. The total amount of carbon source is 5 g/L. Cells grown on 5 g/L glucose or cellobiose were used as the negative or positive control, respectively. Polycellulosomal protuberance structures could be observed on the cell surface of C. thermocellum when 0.5 g/L cellobiose was present in the medium. A scale bar is shown at the bottom right. [file MBT2-11-905-s004.tif]

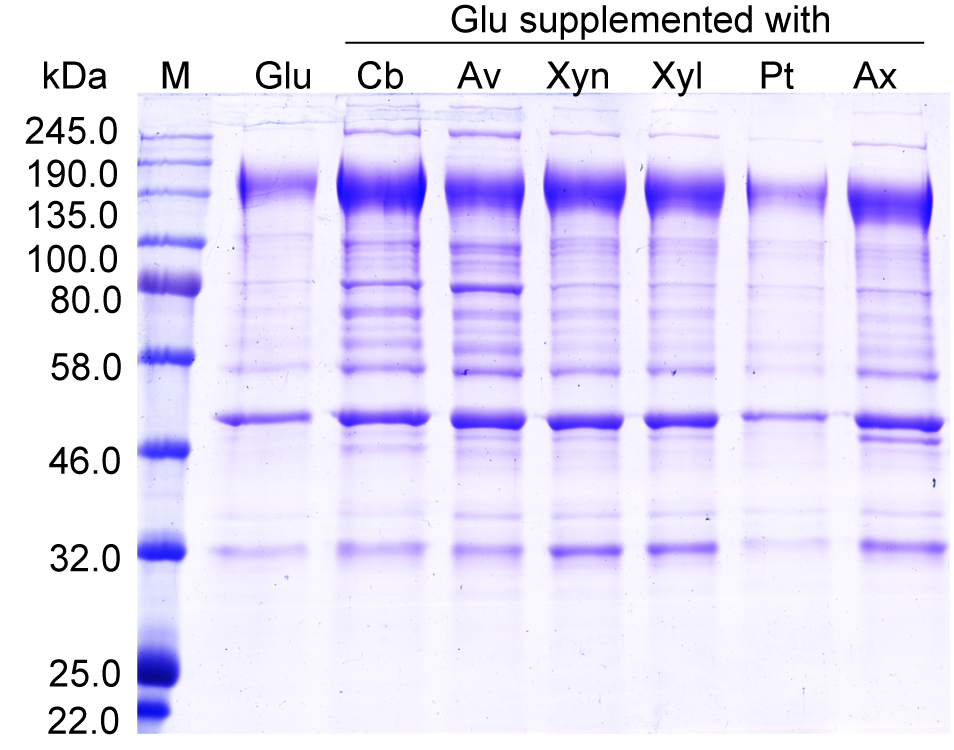

Supplement: Supplementary file 5 — Figure S5. SDS‐PAGE analysis of extracellular proteins of C. thermocellum cultivated with glucose (Glu) as the carbon sources. 0.5 g/L cellobiose (Cb), Avicel (Av), xylan (Xyn), xylose (Xyl), pectin (Pt), or arabinoxylan (Ax) was supplemented as the inducer. M, protein standards. [file MBT2-11-905-s005.tif]
