# Supplementary material for: Inducing effects of cellulosic hydrolysate components of lignocellulose on cellulosome synthesis in Clostridium thermocellum
Source: Microb Biotechnol. 2018 Jun 25;11(5):905–16. doi: 10.1111/1751-7915.13293 (PMC6116742; doi:10.1111/1751-7915.13293)
Supplement: Supplementary file 6 — Table S1. The specific activity, concentration and ScaA proportion of the proteins produced by C. thermocellum cells grown on different carbon sources. [file MBT2-11-905-s006.docx]

**Table S1**. The specific activity, concentration and ScaA proportion of the proteins produced by *C. thermocellum* cells grown on different carbon sources^a^

| Substrate^a^ | S (U/mg)^b^ | | C (μg/mL)^c^ | | | pScaA (%)^d^ |
| --- | --- | --- | --- | --- | --- | --- |
|  | S_e_ | S_c_ | C_e_. | C_cp_ | C_p_ |  |
| Glu | 34.69 ± 5.11 | 77.90 ± 2.88 | 20.01 ± 1.36 | 6.95 ± 0.09 | 175.20 ± 6.82 | 0.90 ± 0.07 |
| Cb | 101.31 ± 3.40 | 176.67 ± 2.85 | 45.05 ± 2.03 | 10.89 ± 1.27 | 327.67 ± 5.31 | 2.94 ± 0.07 |
| Av | 312.94 ± 4.39 | 369.65 ± 26.00 | 78.54 ± 1.54 | 17.99 ± 0.42 | 306.53 ± 11.97 | 7.80 ± 0.54 |
| Inducing experiment | |  |  |  |  |  |
| Glu+Cb | 60.60 ± 0.39 | - | 26.15 ± 3.97 | - | 211.72 ± 11.10 | - |
| Glu+Av | 128.02 ± 11.46 | - | 31.36 ± 1.39 | - | 177.77 ± 4.52 | - |
| Glu+Xyn | 30.05 ± 6.46 | - | 21.74 ± 1.00 | - | 197.14 ± 15.34 | - |
| Glu+Xyl | 40.80 ± 4.04 | - | 16.41 ± 2.92 | - | 189.46 ± 8.32 | - |
| Glu+Pt | 35.23 ± 9.87 | - | 21.19 ± 0.09 | - | 215.24 ± 8.44 | - |
| Glu+Ax | 40.20 ± 2.04 | - | 23.56 ± 0.38 | - | 189.46 ± 9.24 | - |

^a)^ The total amount of the carbon source was 5 g/L. For inducing experiment, 0.5 g/L cellobiose (Cb), Avicel (Av), xylan (Xyn), xylose (Xyl), pectin (Pt), or arabinoxylan (Ax) was supplemented in medium with 4.5 g/L glucose (Glu). Average values and standard errors were calculated from three independent analyses.

^b)^ S indicates the specific activity of the extracellular (S_e_) or cellulosomal (S_c_) proteins

^c)^ C indicates the protein concentration of the extracellular (C_e_), cellulose-affinity purified (C_cp_) or pellet (C_p_) proteins in 100-mL culture.

^d)^ pScaA was calculated based on the extracellular proteins.

-, not detected.
