# Supplementary material for: Inducing effects of cellulosic hydrolysate components of lignocellulose on cellulosome synthesis in Clostridium thermocellum
Source: Microb Biotechnol. 2018 Jun 25;11(5):905–16. doi: 10.1111/1751-7915.13293 (PMC6116742; doi:10.1111/1751-7915.13293)
Supplement: Supplementary file 7 — Table S2. The proportion (%) of the MS identified non‐cellulosomal proteins in extracellular proteins and cellulose‐affinity purified proteins of C. thermocellum cells grown on different carbon sources. [file MBT2-11-905-s007.docx]

**Table S2.** The proportion (%) of the MS identified non-cellulosomal proteins in extracellular proteins and cellulose-affinity purified proteins of *C. thermocellum* cells grown on different carbon sources^a^

|  | Extracellular proteins | | | Cellulose-affinity purified proteins | | |  |
| --- | --- | --- | --- | --- | --- | --- | --- |
|  | Glucose | Cellobiose | Avicel | Glucose | Cellobiose | Avicel | |
| S-layer | 24.7 | 11.6 | 14.6 | 20.7 | 8.7 | 8.9 | |
| CbpB | 3.8 | 13.7 | 1.5 | 7.0 | 5.3 | 3.2 | |
| ADH | 3.8 | ND | ND | 4.1 | 2.4 | 1.6 | |
| CAO | 4.7 | 2.3 | ND | 2.6 | 1.7 | ND | |
| GAPDH | 1.4 | 2.6 | ND | 4.0 | 2.2 | 2.3 | |
| CbpA | 5.3 | 3.2 | 2.3 | 6.4 | 2.6 | 2.4 | |

^a)^ S-layer (S-layer homology protein, Clo1313_RS15300), CbpB (sugar ABC transporter substrate-binding protein, Clo1313_RS06075), ADH (alcohol dehydrogenase, Clo1313_RS09240), CAO (copper amine oxidase-like protein, Clo1313_RS11630), GAPDH (glyceraldehyde-3-phosphate dehydrogenase, Clo1313_RS10615), and CbpA (sugar ABC transporter substrate-binding protein, Clo1313_RS09245). ND, not detectable
